# Transparent Quality Optimization for Machine Learning-Based Regression in Neurology

**DOI:** 10.3390/jpm12060908

**Published:** 2022-05-31

**Authors:** Karsten Wendt, Katrin Trentzsch, Rocco Haase, Marie Luise Weidemann, Robin Weidemann, Uwe Aßmann, Tjalf Ziemssen

**Affiliations:** 1 Software Technology Group, Technische Universität Dresden, 01187 Dresden, Germany; karsten.wendt@tu-dresden.de (K.W.); uwe.assmann@tu-dresden.de (U.A.); 2 Center of Clinical Neuroscience, Neurological Clinic, University Hospital Carl Gustav Carus, 01307 Dresden, Germany; katrin.trentzsch@uniklinikum-dresden.de (K.T.); rocco.haase@uniklinikum-dresden.de (R.H.); marieluise.weidemann@uniklinikum-dresden.de (M.L.W.); robin.weidemann@uniklinikum-dresden.de (R.W.)

**Keywords:** machine learning, inertial measurement units, multiple sclerosis, deep learning, software quality, fractional factorial design benchmark

## Abstract

The clinical monitoring of walking generates enormous amounts of data that contain extremely valuable information. Therefore, machine learning (ML) has rapidly entered the research arena to analyze and make predictions from large heterogeneous datasets. Such data-driven ML-based applications for various domains become increasingly applicable, and thus their software qualities are taken into focus. This work provides a proof of concept for applying state-of-the-art ML technology to predict the distance travelled of the 2-min walk test, an important neurological measurement which is an indicator of walking endurance. A transparent lean approach was emphasized to optimize the results in an explainable way and simultaneously meet the specified software requirements for a generic approach. It is a general-purpose strategy as a fractional–factorial design benchmark combined with standardized quality metrics based on a minimal technology build and a resulting optimized software prototype. Based on 400 training and 100 validation data, the achieved prediction yielded a relative error of 6.1% distributed over multiple experiments with an optimized configuration. The Adadelta algorithm (LR=0.000814, $f_{\mathrm{ModelSpread}}\;=\;5$, $n_{\mathrm{ModelDepth}}\;=\;6$, $n_{\mathrm{epoch}}\;=\;1000$) performed as the best model, with 90% of the predictions with an absolute error of <15 m. Factors such as gender, age, disease duration, or use of walking aids showed no effect on the relative error. For multiple sclerosis patients with high walking impairment (EDSS Ambulation Score ≥6), the relative difference was significant (n=30; 24.0%; p<0.050). The results show that it is possible to create a transparently working ML prototype for a given medical use case while meeting certain software qualities.

## 1. Introduction

Machine Learning (ML)-based approaches for medical use cases and questions are becoming increasingly important and applied in specific medical scenarios. With regard to the prospective impact of such prediction or decision support systems, the quality of the results and the software itself, i.e., the systems’ transparency and transferability for domain experts and admission instances for medical products are import and require reflection during the design process [1]. Result quality, e.g., prediction accuracy or robustness, and software quality, e.g., explainability, modularity or reusability, can be contrary design objectives; hence a trade-off has to be achieved [2,3].

In this work, a Deep Learning (DL) software prototype for a concrete medical use case from the field of neurology was systematically optimized regarding different, previously chosen software qualities and designed for further recoverability and transferability to similar applications.

### 1.1. Background

Multiple sclerosis (MS) is a chronic autoinflammatory demyelinating disease of the central nervous system and the most frequent cause of non-traumatic disability in young adults [4,5]. During disease progression, disseminated inflammatory lesions that spread throughout the central nervous system lead to dysfunction in multiple functional systems responsible for a variety of different neurological deficits [6]. Especially progressive gait impairment and limitation of mobility are some of the most common pathognomic symptoms even in the early stages of the disease and contribute substantially to the loss of patients’ quality of life [5,7]. Gait impairments in people with Multiple Sclerosis (PwMS) are characterized by decreased gait speed, gait endurance, step frequency and cadence, and increased gait variability [8,9]. For early and detailed assessment of increasing mobility limitations in PwMS, the Dresden Protocol of Multidimensional Walking Assessment (DMWA) was implemented as part of routine clinical examination, and various motion analyses using different gait parameters and measurement methods have been performed to assess gait, stance, and balance [10]. Thereby, spatiotemporal gait analysis is performed by using a wireless body-worn sensor system, named as Mobility Lab System (MLS) and the GAITRite system. In this scope, the 2-min walk test (2MWT) is an important ingredient of a structured gait-testing battery, assessing the distance a patient is able to walk during two minutes. To date, the distance walked has been measured manually by assistants using an odometer. The odometer is currently considered the gold standard for measuring walking distance traveled in the clinical setting [11,12]. However, its use also reveals some disadvantages. For one, the odometer is always guided by the rater, which means that the different evasive movements, due to the pathological gait pattern or obstacles in the course, are not taken into account when the patient walks. In addition, rotation at the end of a gait is insufficiently detected because the reversal angle of the odometer is different. A digitized approach with the use of Inertial Measurement Units (IMUs) is increasingly being considered to avoid high inter-rater reliability and to increase the efficiency and accuracy of the measurement process [13,14]. We want to take this approach even further and develop and optimize an automated system for distance measurement by using ML technologies based on the aggregated multidimensional data of PwMS from the MLS.

### 1.2. Motivation for Transparent Optimization Design

Setting up and optimizing an ML-based software prototype to predict medical measurements, in this case the walking distance of PwMS, based on high-dimensional and heterogeneous (aggregated sensor) data implies different quality requirements, based on [15]. The selected qualities from the medical and technical point of view are shown in Table 1.

As the impact of technological and design decision remains unknown until implementation, which is characteristic for experimental data-driven software approaches [3,16], a lean and fast prototyping approach is recommended as a working metastrategy. The following challenges for ML systems [1,2] apply for this use case: (i) evolving leading questions and motivation; (ii) shifting of evaluation strategies and of definitions for result quality and metrics; (iii) changing selection of data and features, technologies, pre- and post-processing steps, and the configuration space; (iv) ongoing ML pipeline optimization strategy to achieve best possible result; and (v) continuous integration of domain expertise.

To cover and track the previously listed qualities and face these challenges, a transparent, and thus recoverable and reusable optimization design approach allows for later explainable changes and adaptations in contrast to one-fits-all or automated black-box solutions [17,18]. In summary, the software optimization and design strategy treats the task as a problem of competitive objectives, i.e., to achieve the best possible proof of concept in given time, as typically the realization resources are limited. In other words, the experimental data-driven software prototype should be meaningful, flexible, lean, extensible, and explainable enough to meet the specified software qualities for the given and prospective use cases.

Hence, this paper investigates the impact of the optimization strategy on the software quality in a quantitative (quality evaluation) and qualitative way (design reflection), and in this way, the medical potential of ML-based approaches for gait analyses. The leading research questions are:

- Is it possible to create working ML-based prediction prototypes for specific medical use cases with only few data of low/medium quality?
- What are the best possible prediction results for these kinds of approaches?
- What are the influencing factors for the quality of medical ML prototypes, especially for prediction quality?

## 2. Methods and Materials

In the following section, the technical and medical state of the art is reflected briefly before the actual ML approach and the according analysis strategy for the result quality are introduced.

### 2.1. State of the Art

#### 2.1.1. Data-Based Prediction Approaches

In contrast to the conventional method to measure the patients’ walking distance manually, i.e., medial staff follows the patient utilizing a distance measurement wheel, the 2MWT distance should be deduced from multi-dimensional sensor data from the MLS. The prediction of such a value can be modeled as a regression problem. First, a large number of conventional regression analysis approaches, e.g., linear or logistic regression exists that influence the result quality as well as the transparency of the model [19]. In particular, for the multi-input scenarios the optimization algorithms have to tune large sets of coefficients to minimize a given target function, which is a complex and potentially extensive task [20].

Furthermore, a large number of techniques from the field of supervised ML [21] can be utilized for prediction tasks, wherein each approach implies different challenges and potentials. ML technology is already applied for medical use cases, e.g., in [22,23]. Hence, and with regard to the complexity of conventional statistical approaches, it is valid to utilize ML as a large technology group for the 2MWT prediction problem.

#### 2.1.2. Software Technologies

Currently, standard software libraries and frameworks for ML are available, which provide a large number of researched techniques and are well-maintained and documented, e.g., TensorFlow [24] or Scikit-learn [25]. The frameworks are mainly based on Python or other established programming languages and are deployable to standard PCs, i.e., they require no special hardware or High Performance Computing (HPC) systems. The appropriate configuration, parametrization as well as the actual pipeline setup for a specific use case are not trivial tasks and have strong impact on the result quality and performance. There exist approaches and solutions for this so called hyper-parameter optimization [26] in the ML frameworks themselves as well as independent solutions, e.g., Optuna [27] or Auto-WEKA [28].

### 2.2. Dataset

The data of 511 PwMS who completed a multidimensional gait analysis as part of their routine clinical examination between June 2018 and February 2019 at the MS Center Dresden of the University Hospital Carl Gustav Carus Dresden were used. To record spatiotemporal gait parameters, all study participants wore six Mobility Lab Opal sensors (APDM, Portland, OR, USA), located on the patient’s wrists, ankles, sternum, and lower back. Each sensor unit contained a three-axis accelerometer, a three-axis gyroscope, and a magnetometer. We used the accelerometer data to estimate the distance walked by the patient. Data from patients with a confirmed MS diagnosis who were able to walk with or without assistive devices were included. Data acquisition was performed according to the guidelines for good clinical practice and was approved by the local ethics committee (BO-EK-320062021).

### 2.3. ML-Based Software Approach

**Figure 1:**
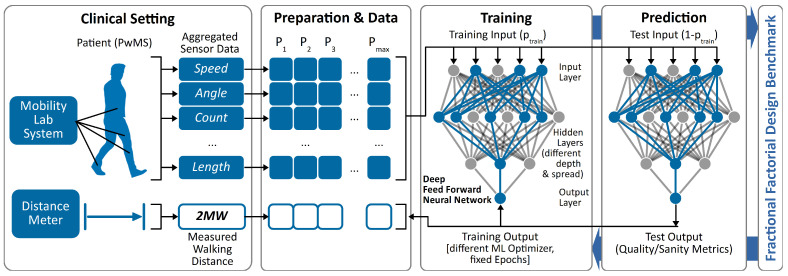
Minimal viable ML-based approach for 2MWT prediction. After aggregating MLS data and manually measured walking distances from PwMS, the data are transposed to a table-based (columns $P_{1..\max}$: patients; rows: speed, …, 2MW: features and learning objective), thus ML-compatible representation, prepared, split and fed into a DFFNN based on TensorFlow. The model training bases on incrementally improved configurations (FFDB) to optimize the prediction quality, expressed by predefined metrics. [Abbreviations: ML = Machine Learning; 2MWT = 2 minute Walk Test; MLS = Mobility Lab System; PwMS = People with Multiple Sclerosis; DFFNN = Deep Feed Forward Neural Network; FFDB = Fractional Factorial Design Benchmark].

The approach is described in Figure 1.

#### 2.3.1. Machine-Learning Setup Design

In this section we describe the ML setup design.

(1) $$MSE_{\mathrm{arr}}\;=\;\frac{\sum_{n_{\exp}}\sqrt{MSE}}{n_{\exp}\cdot dist_{\mathrm{avg}}}$$

Formula (1) defines the Average, Relative, Rooted Mean Square Error ($MSE_{\mathrm{arr}}$) as overall prediction quality metric, with the number of experiments per configuration ($n_{\exp}$) and $dist_{\mathrm{avg}}$ as the global scaling factor, enabling better comparability between usa cases and datasets.

(2) $${\left.w_{\mathrm{rev}}^{i}\;=\;\frac{\sum_{n_{\mathrm{ModelSpread}}}w_{\mathrm{rev}}^{i-1}}{n_{\mathrm{ModelSpread}}}\right|}_{w_{\mathrm{ModelDepth}}=1}$$

Formula (2) describes the Reverse Synapse Weight for Input Features ($RSW_{\mathrm{input}}$) as weight sum of subsequent synapses within the Deep Feed Forward Neural Network (DFFNN) to express the influence of different input neurons, i.e., data features. Reflecting the objective to develop a Minimal Viable Solution (MVS) for the 2MWT with respect to the aspired quality requirements (see Section 1.2), the following general-purpose ML setup as shown in Table 2 was defined.

#### 2.3.2. Setup Optimization

To achieve optimal results, i.e., minimal prediction errors, it is necessary to optimize the predefined ML pipeline. As stated above, the optimization space is defined by four parameters. Assuming multiple runs per configuration, Formula (3) describes an estimation of the optimization space size:

(3) $$\begin{array}{cc} n_{\mathrm{conf}}^{\mathrm{est}} & =n_{\exp}\cdot c_{\mathrm{LR}}\cdot c_{\mathrm{ModelSpread}}\cdot c_{\mathrm{ModelDepth}}\cdot c_{\mathrm{Alg}}\\ \; & =1.26\cdot 10^{5} \end{array}.$$

Hence, even a small *n*_exp_ with *n*_exp_ = 10 and a deliberately small optimization setup (*c*_LR_ = 50, *c*_ModelSpread_ = *c*_ModelDepth_ = 6, *c*_Alg_ = 7) lead to a large number of training runs to tune and evaluate the approach, which exceeds the capacity of a normal PC. Because the application of (hyper-) optimization frameworks is a complex and also errorprone process [27,30] and an automated-tuned configuration may reduce the explainability and recoverability of the approach, a Fractional Factorial Design Benchmark (FFDB) as described in Table 3 was designed. First, the number of training iterations (*n*_epoch_) is set to a sufficiently large value as it intersects with the *LR*. Subsequently, a mid-size DFFNN model is chosen (*f*_ModelSpread_ = *n*_ModelDepth_ = 3) and the optimal *LR* is determined for each TensorFlow training algorithm (|*t*_alg_| = 7) with regard to the defined quality metric (see Formula (1)). *f*_ModelSpread_ is a factor to describe the size of the hidden layers in dependence of the size of the input vector and *n*_ModelDepth_ describes the number of hidden layers. Defined by these spread and depth parameters, models of different sizes are optimized by the training algorithm, utilizing the former detected optimal *LR*. Finally, each set of model and algorithm is compared to detect the optimal configuration for the 2MWT and the chosen technology.

### 2.4. Analysis Strategy for the Prediction Quality

To analyze the defined prediction quality and its dependencies, $MSE_{\mathrm{arr}}$ (1), as well as its SD are evaluated repeatedly ($n_{\exp}$) after each step of the FFDB. Furthermore, a moving average ($size_{\mathrm{windows}}=3$) is utilized to highlight the overall course of the charts. For each input feature of each trained model, the $RSW_{\mathrm{input}}$ (2) is calculated to illustrate the features’ impact on the overall prediction. The runtime of the model training as well as of the execution is considered as not relevant for the experimental character of the approach, and thus, not evaluated. The dependencies are shown in Section 3.2, including line charts for the LR dependency per algorithm, heat maps for model size dependencies, bar charts for the final algorithm comparison, aggregated histograms and scatter plots for the best models’ prediction and a table of the top-5 features with large positive and negative overall impact for the sanity check.

### 2.5. Reliability and Validity of the Optimized Algorithm

To further determine the reliability and the validity of the optimized algorithm, we estimated the Intraclass correlation coefficient (ICC) [31] between the initially measured and the predicted values and searched for factors that may be associated with differences in the algorithm’s precision. Therefore, we compared relative differences in relation to gender, age, disease duration, overall and specific disabilities (via EDSS) [32] and the use of walking aids (fixed effects) by applying a linear mixed model analysis that also included the assessing staff member as random factor. A *p*-value of <0.05 is considered to indicate significant differences. Estimates are reported as mean ± SD. Absolute and relative differences are calculated as absolute values.

## 3. Results

The algorithms were evaluated with 511 measurements of PwMS. For a more detailed analysis comparing results of the best performing algorithm to the directly measured walking distance between subgroups, measurements of 455 PwMS were available. Overall, 67% were female, enrolled at an age of 43.17±11.57 and with a median Expanded Disability Status Scale (EDSS) score of 2.5. Walking aids were used by 7.7% of PwMS. The average performance in the timed 25-foot walk was 6.26±3.37 s [31]. In the following, the ML/DL software setup as well as the results of the generic FFDB for the concrete medical use case are presented.

### 3.1. Software Execution

The accumulated sensor data of each patient (n=511) was collected manually, imported and prepared as CSV file as described in Section 2.3.1, i.e., the features were selected, formatted, and normalized, as well as missing items or outliers were treated. Finally, for each experiment (training run), the data was randomly split up into a training (80%) and a validation set (20%), and transposed to a technologically appropriate input format. Utilizing the ML software technology TensorFlow, a DFFNN as a regressor of variable sizes was designed, which takes 92 transposed MLS features as input and were trained against the manually measured walking distance utilizing different ML algorithms. The measured average walking distance was $dist_{\mathrm{avb}}=137.42$ m, serving as a scaling factor for the $MSE_{\mathrm{arr}}$ (see Formula (1)).

The parameter optimization was performed by an FFDB as described in Section 2.3.2. The experiments were executed on a Lenovo Working Station P52 several times, while collecting quality measurement data and ollowing the defined analysis strategy (see Section 2.4). Finally, after completing the FFDB, an optimized ML pipeline was available to predict the walking distances of PwMS based on aggregated sensor data.

### 3.2. Fractional Factorial Benchmark Results

In the following, the quality of the intermediate and final quality measurements during the FFDB (see Table 3), are shown.

#### 3.2.1. Definition of the Number of Epochs

To ensure an appropriate training time and decouple to dependent *LR*, the FFDB starts by defining *n*_epoch_ = 1000, as the error-reduction plots of different training algorithms shows (not presented here), that even for comparatively small *LR*s this number is sufficient. Runtime is not an issue by definition.

#### 3.2.2. Learning Rate Optimization

**Figure 2:**
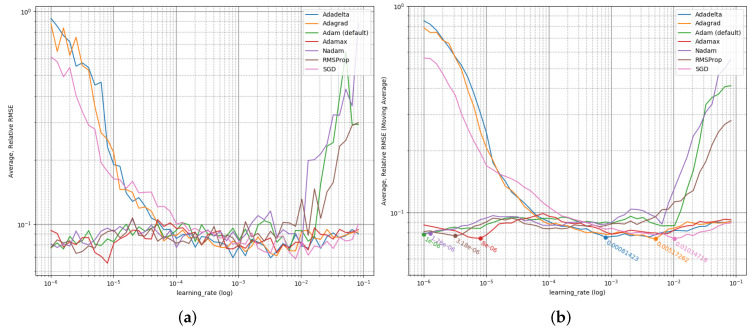
LR Optimization. $MSE_{\mathrm{arr}}$ as prediction quality ((**a**) normal, (**b**) moving average and marked minima) in dependence of LRs for 7 ML training algorithms, optimizing DFFNNs of fixed shape [Abbreviations: LR = Learning Rate, ML = Machine Learning, DFFNN = Deep Feed Forward Neural Network].

Figure 2 shows the prediction qualities’ dependency of the LRs for different training algorithms. It is clearly visible that the algorithms behave differently, SGD, Adagrad, and Adadelta expose high error rates ($MSE_{\mathrm{arr}}=10^{0}$ corresponds to 100% total error (see Formula (1)) for high *LR*s, RMSProp, Adam, and Nadam for low LRs, only Adamax is stable in dependence of the LR. All expose significant noise, despite of multiple experiments per configuration. Hence, it is crucial to determine appropriate LRs individually for each algorithm, which is done based on the moving average.

#### 3.2.3. Model Optimization

**Figure 3:**
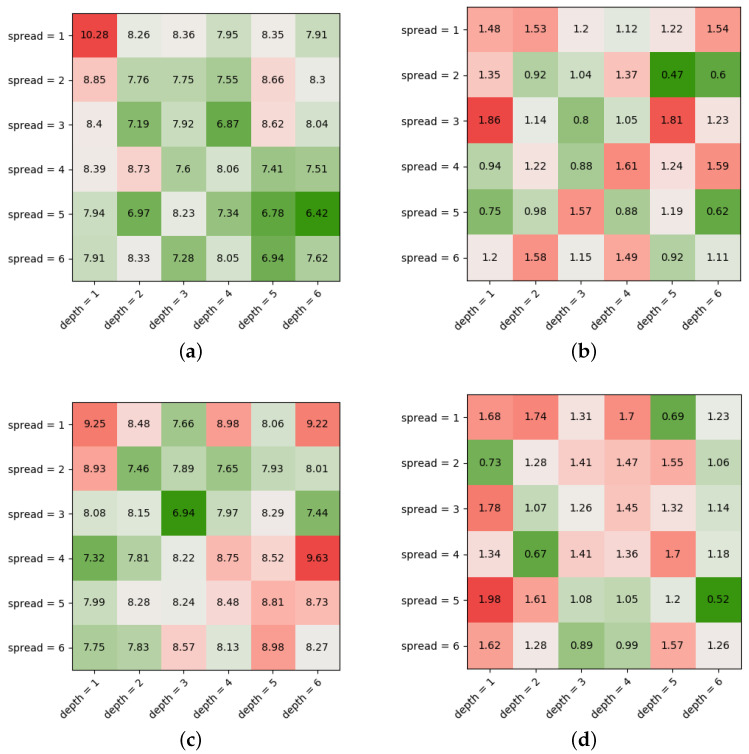
Model Shape Optimization. (**a**) Optimizer = Adadelta; Average, Relative RMSE for Model Spread and Depth (%); (**b**) Optimizer = Adadelta; Average, Relative SD for Model Spread and Depth (%); (**c**) Optimizer = RMSProp; Average, Relative RMSE for Model Spread and Depth (%); (**d**) Optimizer = RMSProp; Average, Relative SD for Model Spread and Depth (%) $MSE_{\mathrm{arr}}$ as prediction quality (**c**) and its SD (**d**) in dependence of $f_{\mathrm{ModelSpread}}$ and $n_{\mathrm{ModelDepth}}$ for the Adadelta (best case) and the RMSProp algorithm (worst case); values are scaled for better readability [Abbreviations: RMSE = Rooted Mean Square Error; SD = Standard Deviation].

Figure 3 shows the prediction qualities’ dependency of the model shape, i.e., $f_{\mathrm{ModelSpread}}$ and $n_{\mathrm{ModelDepth}}$ of the DFFNN for the Adadelta algorithm. The heat maps show different prediction qualities and again a significant noise between the experiments, similar to the LR dependencies, but a minimum for the model shape (5,6), which can be considered as optimal model for this algorithm with optimized LR. The Figure 3 shows the same for the RMSProp algorithm as the worst-performing algorithm. The evaluation was executed for every ML optimization algorithm, but only two results are shown here exemplarily. To summarize, the model shape dependency expose a volatile picture for the prediction quality, with many insufficient model configurations and individual optimal model shapes for each algorithm.

#### 3.2.4. Optimization Algorithm Comparison

**Figure 4:**
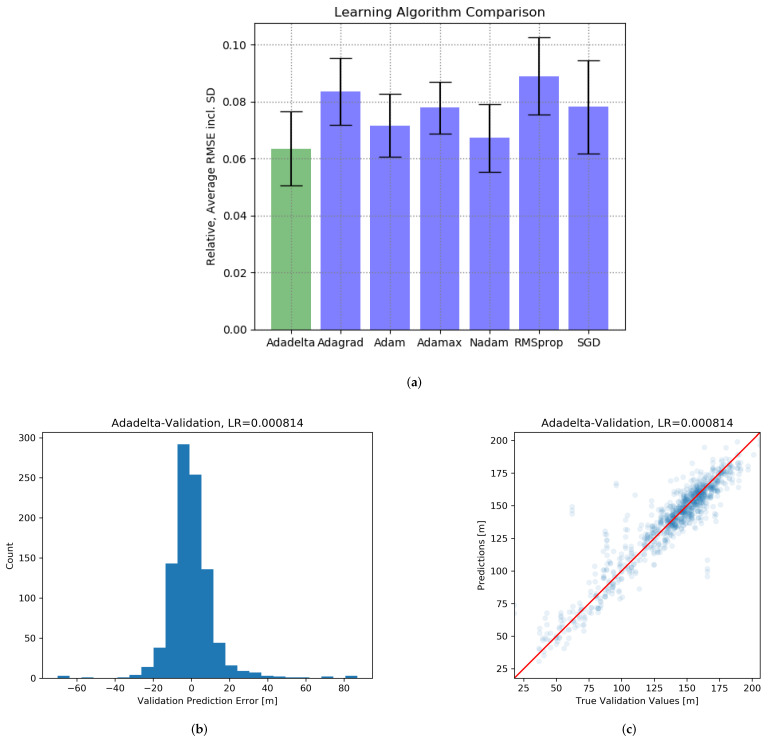
(**a**) Optimization algorithm comparison. $MSE_{\mathrm{arr}}$ as prediction quality and its SD for each algorithm; (**b**,**c**) Prediction distribution; individual results of the best regressor model after completing the FFDB as total error [Abbreviations: SD = Standard Deviation; FFDB = Fractional Factorial Design Benchmark; LR = Learning Rate].

Finally, the quality performance of each algorithm with an optimized configuration can be compared as shown in Figure 4a. On the one hand, it illustrates the Adadelta algorithm is best candidate, yielding an $MSE_{\mathrm{arr}}$ of 0.061 (an average relative error of 6.1%), which corresponds to a total average error of 8.37 m. On the other hand, despite multiple experiments per configuration, all algorithms show a significant SD between 0.009 and 0.016, i.e., single predictions of individual models are volatile and thus, difficult to compare.

#### 3.2.5. Best Prediction Result

Examining the results of the best model (Adadelta algorithm, *LR* = 0.000814, *f*_ModelSpread_ = 5, *n*_ModelDepth_ = 6, *n*_epoch_ = 1000), a histogram and a scatter plot reveals the aggregated predictions’ distribution for validation data for *n*_exp_ = 10 experiments as shown in Figure 4b,c. The distribution indicates that 90% of the predictions expose an absolute error of <15 m; only 3% should be regarded as outliers (bsolute error > 25 m).

#### 3.2.6. Sanity Check

To increase the transparency and the explainability of the approach, the $RSW_{\mathrm{input}}$ was calculated for each input feature of the original data, utilizing the best-performing model (see also Formula (2)). Table 4 shows the top-5 and last-5 feature names in combination with the average, unscaled reverse weight. Features with positive reverse weights imply a large impact of the predicted walking distance, negative value imply a low impact. With respect to the meaning of the feature names, i.e., Cadence, Gait Speed, Stride Length, and Arm Motion, Step Duration, Swing, and Terminal Double Support, respectively, the model appears to act meaningfully and can be considered as a supporting tool for medical treatment.

### 3.3. Reliability Check

In 455 PwMS, the initially measured distance was 138.06±33.37 m, and the average predicted distance was 137.85±32.28 m. This leads to a mean individual difference of 7.40±8.77 m and a respective relative difference of 6.8±16.7%. An excellent overall reliability was achieved Intraclass correlation coefficient (ICC) = 0.942, 0.930–0.951 95% confidence interval). No influence in relative differences could be detected for gender (p=0.569), age (p=0.122), disease duration (p=0.086), the use of walking aids (p=0.278), overall disability (p=0.610), or most specific disabilities (visual: p=0.445; brainstem: p=0.491; pyramidal: p=0.930; cerebellar: p=0.115; sensory: p=0.095; bowel & bladder: p=0.332; cerebral: p=0.071). Only in cases of increased impairment in ambulation (EDSS ambulation score ≥6) the relative difference was increased (n=30; 24.0%; p<0.050 in all pairwise comparisons).

## 4. Discussion

The distance achieved during gait endurance tests serves as an important indicator of walking ability and physical performance [32]. To date, there is no general automatic measurement approach to determine the actual distance walked after completion of the 2MWT, although numerous spatial and temporal gait parameters can be extracted. Digitization in this area through the use of IMUs is increasingly being used to determine gait disturbances [33,34,35] and supports more sensitive patient monitoring. Following this approach, we provide a proof of concept in this study for the application of state-of-the-art ML technology for comparatively little data to predict an important neurological measurement for a specific use case. With regard to the research questions in Section 1.2, this concept reflects the previously specified software qualities and thus, contributes to a general purpose and lean approach for similar use cases, utilizing standardized quality metrics. The solution shows that it is possible to achieve good result qualities (here prediction accuracy) even for small datasets, which are typical for medical use cases. Hence, it is not necessary, and often not possible, to build on big data solutions, but very specific optimization is required. In this case, this issue was solved by a FFDB to fine tune the optimization parameters in a transparent and recoverable manner. The achieved prediction yielded a relative error of 6.1% and a fraction of 3% for outliers basing on 400 training and 100 validation data items (patients), distributed over multiple experiments with an optimized configuration. With regard to the number and the quality of the data items, this is a viable result, enabling the approach for practical use. Referring to the high SD across multiple experiments, it is recommended to set up multiple DFFNN in parallel and calculate the average prediction for productive usage. In detail, the prediction quality could be improved significantly by changing the training (model optimization) algorithm and the according LR, as well as the model structure itself ($f_{\mathrm{ModelSpread}}$, $n_{\mathrm{ModelDepth}}$). This means that the default selection and parameters were not optimal and even for small ML/optimization problems of this kind a strategic setup was necessary to tackle the large configuration space in an efficient manner. The chosen FFDB can be regarded as a compromise between global optimization, time efficiency, and transparency, as extensive grid searches would have consumed much more computation time or required another hardware setup, and hyper optimization frameworks like Keras Tuner or Optuna are extensive to adapt to the use case and their results are harder to explain to the domain stakeholders. To provide qualities like reliability and robustness, average standard variation metrics were evaluated for each configuration, which was tested during multiple experiments with different random seeds, influencing the training and validation data split and the starting configuration of the models. It turned out, that for optimized LRs the average, relative SD stayed below 2% for multiple model instances, which stands for a good reliability and also allows deducing robust behavior for new data. The algorithm achieved an excellent overall reliability and only provided less accurate results in cases of PwMS with severe limitations in ambulation. This limitation may be overcome with further datasets that include a larger portion of more severely impaired PwMS. The decision against automated hyper-optimization techniques, but for a FFDB and a final sanity check also contributed to explainability and transparency, as this explicit white-box approach clarifies the decision path toward the optimized software prototype. In this way, the optimization strategy is descriptive in a step-wise manner and allows dedicated subsequent changes to adapt the solution to new data or other questions. Furthermore, the descriptive strategy enables the experiments’ repetition at arbitrary points within the FFDB decision path, i.e., it ensures recoverability and points out the factors’ influence on the result. As the prototype was for experimental use for domain experts, in this case medical staff, the accessibility was provided by a single configuration access point, describing the ML pipeline steps as well as its configuration, or the search space, respectively. In this way, the use case specific configuration is separated from the prototype itself, which allows its reusability, i.e., its application and adaptation to new, similar use case. Finally, the interoperability was ensured by the selection of platform independent software technology, i.e., no special hardware setup is required to execute the prototype. As the ML pipeline structure followed technological standards, the functional modules were established, enabling modularity. With regard to the ease of use for medical experts, leanness was focused during the software design and achieved by simple structures and minimal functional coverage. On the other side, the evaluated data was small in comparison to other studies. If more data become available, the study should be repeated with the same optimization strategy to make the results even more reliable and to evaluate the influence of the data size on the results. The data was provided in an aggregated form, i.e., the raw data could improve the prediction quality even further, but would imply a much larger data size, and thus, a different data handling and potentially other ML models. With regard to data complexity, i.e., the large dimensionality, the selection and weighting of the data feature were not finally investigated, i.e., feature redundancies or optimized weighting remain not reflected. Furthermore, other ML models or libraries could yield other or better results (quality, reliability), but were not investigated with respect to the other desired, but contrary software qualities (e.g., explainability, accessibility, leanness). The same applies for in-depth parameter optimization for the chosen training algorithms.

## 5. Conclusions

From a medical point of view, this study shows how technological advances present the opportunity to complete preexisting technique and clinical assessments. Thus, further clinical usage and more objective results support more sensitive progression monitoring and clinical decision making. The results show that it is possible to create a transparent and working ML prototype for a given medical use case while simultaneously meeting specific software qualities. The selected optimization design yielded good and reliable prediction results, while the other software qualities ensured the transferability to similar problems and transparency for the domain stakeholders. It was shown that the strategy of previously selected and tracked software qualities for the specific domain in combination with a FFDB to gradually approach a result optimum and a minimal set of ML/DL technology leads to a lean and well-explained software prototype with low technical requirements and minimal access restrictions for the domain experts. In this way, an optimization design for potentially more ML software systems for similar application fields was contributed. Consequently, data-driven monitoring of disability progression reaches a new landmark with the chance to determine objective insights into personalized patient gait performances more precisely and faster in the field of neurology [36].

## Figures and Tables

**Table 1:** Aspired software qualities for ML prototype; ML = Machine Learning.

Quality	Description
Prediction quality	The results should be as good as achievable
Reliability	A statement about the results’ steadiness should be available
Robustness	The results should be tolerant w.r.t. new or other data
Transparency, explainability	The prediction approach should be as transparent and explainable as possible w.r.t. the selected ML technologies
Recoverability	The setup, as well as the result should be recoverable
Accessibility	The prototype should be usable by physicians
Interoperability, modularity, reusability	The prototype should not be restricted to specific software technologies and designed in a way to allow functionality replacement or the adaption to other (medical) use cases
Leanness	The prototype should base on a small specific code base to reduce dependencies and achieve the result as fast as possible

**Table 2:** ML Software Setup [Abbreviations: ML = Machine Learning; GPU = Graphics Processing Unit; HPC = High-Performance Computing; DFFNN = Deep Feed Forward Neural Network; SD = Standard Deviation; LR = Learning Rate].

Aspect	Description
Technical environment	PC with sufficient hardware; no grid of GPU or HPC system
Data format requirements	Table based, e.g., CSV format
Data import	Use case specific; manual import; standard normalization and error handling
ML technology	TensorFlow [24]; no hyperparameter optimization framework
Model	DFFNN [29] of different shapes as regressor
Quality metrics	$MSE_{\mathrm{arr}}$ based on [19], see Formula (1); SD of $MSE_{\mathrm{arr}}$
Sanity check	$RSW_{\mathrm{input}}$, see Formula (2)
Result optimization objective	Minimize MSE, $MSE_{\mathrm{arr}}$ respectively
Optimization space	LR, $f_{\mathrm{ModelSpread}}$, $n_{\mathrm{ModelDepth}}$, $t_{\mathrm{alg}}$

**Table 3:** Fractional Factorial Design Benchmark. Order and context of tuned parameters to optimize. the prediction quality in explainable and recoverable manner [Abbreviations: LR = Learning Rate; ML = Machine Learning].

Factor	Actions
*n*_epoch_ = 1000	Small initial *LR* (<default) test all ML optimization algorithms fixed: *LR*, *f*_ModelSpread_, *n*_ModelDepth_
LR = 10^−6^..10^−1^ (exp. step size)	Increase *LR* step-wisely test all ML optimization algorithms with different *LR* fixed: *n*_epoch_, *f*_ModelSpread_, *n*_ModelDepth_
*f*_ModelSpread_ = 1..6 *n*_ModelDepth_ = 1..6	Increase *f*_ModelSpread_ and *f*_ModelSpread_ step-wisely test all ML optimization algorithms for different model sizes fixed: *n*_epoch_, *LR*
*t*_alg_	Test all ML optimization algorithms fixed: *n*_epoch_, *LR*, *f*_ModelSpread_, *n*_ModelDepth_

**Table 4:** Sanity check. $RSW_{\mathrm{input}}$ as impact indicator for top-5 and last-5 features [Abbreviations: R = right, L = left, GCT = Gait Cycle Time].

Feature	Reverse Weight
Lower Limb - Cadence R (steps/min)	0.148015271
Lower Limb - Gait Speed R (m/s)	0.130966869
Lower Limb - Gait Speed L (m/s)	0.103930242
Lower Limb - Stride Length L (m)	0.096930698
Lower Limb - Cadence L (steps/min)	0.092576412
…	…
Upper Limb - Arm Range of Motion L (degrees)	−0.059278792
Lower Limb - Step Duration L (s)	−0.067239581
Lower Limb - Swing L (%GCT)	−0.067780426
Lower Limb - Terminal Double Support L	−0.069253672
Lower Limb - Double Support L (%GCT)	−0.083872649

## Data Availability

The data presented in this study are available on request from the corresponding author. The data are not publicly available due to patient confidentiality.
